# Extracts of *Andrographis paniculata* (Burm.f.) Nees Leaves Exert Anti-Gout Effects by Lowering Uric Acid Levels and Reducing Monosodium Urate Crystal-Induced Inflammation

**DOI:** 10.3389/fphar.2021.787125

**Published:** 2022-01-10

**Authors:** Eldiza Puji Rahmi, Endang Kumolosasi, Juriyati Jalil, Fhataheya Buang, Jamia Azdina Jamal

**Affiliations:** ^1^ Pharmacy Program, Faculty of Medicine, Universitas Pembangunan Nasional Veteran Jakarta, Jakarta, Indonesia; ^2^ Drug and Herbal Research Centre, Faculty of Pharmacy, Universiti Kebangsaan Malaysia, Kuala Lumpur, Malaysia

**Keywords:** andrographolide, xanthine oxidase, uricosuric, pro-inflammatory cytokines, MyD88, NLRP3

## Abstract

*Andrographis paniculata* (Burm.f.) Nees has been found to have anti-inflammatory and immunostimulatory effects. This study was to investigate antihyperuricemic and anti-inflammatory effects of *A. paniculata* leaf extracts. Andrographolide, 14-deoxy-11,12-didehydroandrographolide, and neoandrographolide were quantified in 80% ethanol (EtOH80) and water extracts using High Performance Liquid Chromatography (HPLC) analysis. Antihyperuricemic activity was evaluated using a spectrophotometric *in vitro* inhibitory xanthine oxidase (XO) assay. The most active extract and andrographolide were further investigated in a hyperuricemic rat model induced by potassium oxonate to determine serum uric acid levels, liver XO activity, followed by Western blot analysis for renal urate transporter URAT1, GLUT9, and OAT1 to investigate the excretion of uric acid *via* kidney. Anti-inflammatory activity was assessed by *in vitro* interleukin assay for interleukin (IL-1α, IL-1β, IL-6, IL-8), and tumor necrosis factor (TNF-α) in monosodium urate (MSU) crystal-induced human fibroblast-like synoviocyte (HFLS) cells using ELISA-kits, followed by Western blot analysis for the expression of MyD88, NLRP3, NF-κB p65, and caspase-1 proteins to investigate the inflammation pathway. *In vivo* assay of the most active extract and andrographolide were performed based on the swelling rate and inhibition of pro-inflammatory mediator release from synovial fluid of a rat knee joint induced by MSU crystals. The results showed that the EtOH80 extract had a greater amount of andrographolide (11.34% w/w) than the water extract (1.38% w/w). In the XO inhibitory activity, none of the samples exhibited greater than 50% inhibition. However, in a rat model, EtOH80 extract (200 mg/kg/day) and andrographolide (30 mg/kg/day) decreased serum uric acid levels and reduced liver XO activity, reduced the protein expression levels of URAT1 and GLUT9, and restored the decrease in OAT1 levels. In the *in vitro* anti-inflammatory study, EtOH80 extract and andrographolide significantly decreased production of IL-1α, IL-1β, IL-6, and TNF-α, as well as inhibited the synthesis of MyD88, NLRP3, NF-κB p65, and caspase-1 in a concentration-dependent manner, almost comparable to dexamethasone. The EtOH80 extract (200 mg/kg/day) and andrographolide (30 mg/kg) significantly decreased swelling rate and IL-1α, IL-1β, IL-6, and TNF-α in the synovial fluid of rat models in a time-dependent manner, comparable to indomethacin (3 mg/kg/day). In conclusion, the findings show that EtOH80 extract has a substantial anti-gout effect by lowering uric acid levels and suppressing pro-inflammatory mediator production due to the andrographolide content, that might be beneficial in the treatment of gouty-inflammation.

## Introduction

*Andrographis paniculata* (Burm.f.) Nees (The Plant List, 2021) (Acanthaceae), also known as “King of Bitter,” sambiloto (Indonesia) or hempedu bumi (Malaysia), has long been used in traditional medicine to treat respiratory diseases, skin infections, herpes, dysentery, fever, sore throat, lower urinary tract infections and diarrhea, as well as to reduce inflammation (Jarukamjorn and Nemoto 2008). It is also used to treat snake bites, insect bites, diabetes, and malaria (Burkill et al., 1966). Many medicinal plants, including *A. paniculata*, have been used as spices or food for thousands of years. *A. paniculata* is widely consumed in Indonesia as *Jamu* (herbal drink) and *Lalapan* (fresh vegetable) (Putra, 2003).

Pharmacological activities such as antiplatelet aggregation activity, immunomodulatory activity, hepatoprotective activity, cytotoxicity against cancer cells, anti-inflammatory and antiangiogenic activities, antimalaria, antidiabetic, cardiovascular activity, and antivenom activity have been reported for *A. paniculata* (Puri et al., 1993; Melchior et al., 1997; Deshpande et al., 2014). Diterpene lactones, such as andrographolide, neoadrographolide, and 14-deoxy-11,12 didehydroandrographolide, are active phytochemicals found in *A. paniculata* (Thisoda et al., 2006; Ren et al., 2008). Andrographolide, a major active constituent, is responsible for the most of pharmacological effects, including anti-inflammatory, antibacterial, antitumor, antidiabetic, antimalaria, and hepatoprotective properties (Jarukamjorn and Nemoto 2008). Other diterpene lactones (neoandrographolide and 14-deoxyandrographolide), flavonoids, quinic acids, and xanthones are also mentioned as having a significant effect.

*A. paniculata* extract has been shown to reduce inflammation by inhibiting the expression of iNOS, TNF-α, IL-1β, IL-6, and IL-12, as well as NO production, through the downregulation of p38MAPKs signaling pathways (Liu et al., 2008; Wang et al., 2013). Andrographolide potently modulated the level of LPS-induced TNF-α, IL-6, IL-1β, and IL-10 secretion in human blood in a concentration-dependent manner by selectively down-regulating cytokines and cytokine receptors (TNFSF14, TNF, TNFRSF6, and IL1A), chemokines (CCL8 and CXCL11), JAK/STAT signaling (JAK3 and STAT5A), TLRs family (TLR4 and TLR8) and nuclear factor kappa B (NF-κB) (Parichatikanond et al., 2010).

Salim et al. (2014) discovered that the methanol extract of *A. paniculata* and andrographolide inhibited IL-1α, an important regulatory cytokine whose release after an injury can activate transcription factors NF-kB and activator protein (AP-1), promoting expression of genes involved in cell survival, proliferation, and angiogenesis (Wolf et al., 2001). To our knowledge, there is limited information about the action of *A. paniculata* on gouty arthritis, such as its effects on lowering uric acid levels and MSU-induced inflammatory in synovial cells.

Gout is an ancient inflammatory arthritis that has been documented for thousands of years. The pathogenesis of gout is started with the high level of uric acid in blood and the deposition of MSU crystals in the joint. MSU crystal is a very important factor for gouty inflammation as a pro-inflammatory stimulus by stimulating cells via toll-like receptor signaling (Cronstein and Terkeltaub 2006). MSU crystals can interact with almost all of the synovial cell types including neutrophils, monocytes/macrophages and fibroblast-like synoviocytes. In monocytes, microcrystals stimulate the synthesis of pro-inflammatory cytokines, such as interleukin-1 (IL-1), IL-6, IL-8, tumor necrosis factor alpha (TNF-α) and prostaglandin E_2_ (Pauliot et al., 1998). Previous study on MSU-induced cell activation showed that MSU deposition in the joints was often caused by leucocyte infiltration that leads to inflammation. Long-term inflammation would result in *tophy*, deformation of joints and thickening of synovial walls (Shi et al., 2012).

Interleukin (IL)-1β has been reported to have an important role in MSU-induced inflammation in gout. Thus, the inhibition of IL-1β is able to downregulate the inflammatory responses related to gout (Shi et al., 2012; Malawista et al., 2011). MSU crystals also up-regulate IL-6 and TNF-α secretion when they interact with cells, especially monocytes (Busso and So 2010). TNF-α has been reported to play a complex rote in joint inflammation and tophi formation (Ansari et al., 2021). Moreover, chemokine such as IL-8 (CXCL8) and cyclooxygenase (COX)-2 also play an important role in gouty inflammation (Choi et al., 2005). Prostaglandin E_2_ (PGE_2_) is an eicosanoid that is, biosynthesised from arachidonic acid precursor by the action of cyclooxygenase-2 (COX-2) enzyme and PGE_2_ synthase in endothelial cells.

Based on this information, it is hypothesized that *A. paniculata* extract maybe useful as an anti-inflammatory and anti-hyperuricemic agent in the treatment of gouty arthritis. Thus, the aim of this study was to determine the anti-hyperuricemic effect of *A. paniculata* leaf extracts by exploring the xanthine oxidase (XO) inhibitory and uricosuric effects, asl well as the anti-inflammatory effect on the release of pro-inflammatory markers via the MyD88, NLRP3, NF-κB p65 and caspase-1 pathways in MSU-induced HFLS.

## Materials and Methods

### Chemicals and Reagents

All analytical grade solvents and HPLC-grade acetonitrile were purchased from Merck (Darmstadt, Hesse, Germany) while pure water for HPLC was obtained from ultrapure water system machine (PureLab, United States). Andrographolide (AP, 99.7%), 14-deoxy-11,12-didehydroandrographolide (DAP, < 99.6%) and neoandrographolide (NAP, ≥ 95%) were HPLC grade and acquired from Chromadex (California, United States). Dexamethasone was purchased from CCM Duopharma Biotech Bhd. Xanthine, Tween 20, potassium oxonate, 20 mM HEPES, FBS, pen-strep, RPMI-1640 medium containing L-glutamine, MTT, LPS from *Salmonella abortusequii*, uric acid, DMSO, and tryphan blue^®^ were purchased from Sigma-Aldrich (Missouri, United States). Synoviocyte growth medium was procured from Cell Applications (California, United States). 100× Halt Protease and Phosphatase Inhibitor Cocktail were purchased from Thermo Fisher Scientific (Massachusetts, United States). Xanthine oxidase from bovine milk (20 U/mL) was purchased from Roche Diagnostic GmbH (Mannheim, Baden-Württemberg, Germany). Lymphoprep was purchased from Axis-Shield PoC AS (Oslo, Norway). Phosphate buffer saline (PBS) was purchased from MP Biomedicals (USA). Uric acid and xanthine oxidase kits were purchased from BioVision (Milpitas, California, United States). The concentration of cytokine was tested using appropriate ELISA kits for human. IL-8 ELISA kits were purchased from Abnova, Germany, while all other kits were purchased from Cayman, USA. Limit of detection of these kits were: 2.0 ρg/mL for IL-8, 7.8 ρg/mL for IL-6, and 3.9 ρg mL for IL-1α, IL-1β, and TNF-α. IL-1α, IL-1β, IL-6, and TNF-α in rats were measured using a multi-cytokine bead array detection system (ProcartaPlex, eBioscience, United States).

### Preparation of *Andrographis paniculata* Extracts

*Andrographis paniculata* was cultivated at Field 2 Universiti Putra Malaysia (UPM) in Serdang, Selangor. A voucher specimen (No. SK965/04) was deposited at the Herbarium of the Laboratory of Natural Products, Institute of Bioscience, UPM. The leaves were dried in an oven dryer at 40°C for 3 days before being ground in an electric grinder. Dried powder was extracted with ethanol (80%) at a 1:10 ratio by rigorous maceration for 3 days at room temperature and repeated five times. To eliminate any remaining organic solvent, the organic filtrates were collected and concentrated under reduced vacuum pressure. The solvent-free extract was mixed with water and then freeze-dried (Labconco’s FreeZone 4.5 L Freeze Dry Systems) to get the crude ethanol 80% (EtOH80) extract. Meanwhile, the water extract was obtained from the UPM laboratory of Prof. Dr. Johnson Stanslas. All crude extracts were kept at 4°C until further usage.

### Animals

Male Sprague-Dawley rats (6–8 weeks old, 200–300 g) were procured from Universiti Kebangsaan Malaysia’s Laboratory Animal Resource Unit (LARU-UKM). The animals were kept in plastic cages on a 12 h/12 h light/dark cycle. The temperature and relative humidity were maintained at 25 ± 2°C and 50%, respectively. They were fed a commercial laboratory diet and were given access to food and drink ad libitum during the study. They were given 1 week to acclimate to their surroundings before the experiment. All procedures followed the A CIOMS Ethical Code for Animal Experimentation (Howard-Jones, 1985) and were approved by the Universiti Kebangsaan Malaysia-Animal Ethics Committee (approval reference: FF/2016/JAMIA/27-JULY/772-AUG.-2016-JULY.2019).

### Quantitative Analysis of *Andrographis paniculata* Extracts Using HPLC

Phytochemical analysis of ethanol 80% (EtOH80) and water extracts of *A. paniculata* leaves was carried out using RP-HPLC based on a slightly modified method described by Xu et al. (2008) to quantify the amount of chemical markers, namely andrographolide (AP), 14-deoxy-11,12-didehydroandrographolide (DAP), and neoandrographolide (NAP). The EtOH80 and water extracts were individually prepared by dissolving 10 mg of crude extracts in 1 ml of HPLC-grade methanol, while the chemical markers solution was made by mixing 200 g of each of the three standard diterpene lactones in 1 ml of HPLC-grade methanol. Before analysis, all extract and standard solutions were filtered through a 0.45 m Millipore Millex PTFE membrane. Separate analyses of the extract and standard solutions were performed under the following conditions; column: reversed phase, C-18 column (250 mm × 4.6 mm i.d., 5 µm, Xbridge, Waters, Ireland), detector: PDA (Waters 2998), wavelength: 205 nm, flow rate: 0.8 ml/min, mobile phase: A. methanol, B. water isocratically eluted with 55% A for 30 min with a 10-min equilibration period before injection. The injection volume of the solution was 10 µL.

### Determination of Anti-Hyperuricemic Effect

#### *In Vitro* Xanthine Oxidase Inhibitory Assay

XO inhibitory activity of the extracts (400 μg/ml), chemical markers (100 μg/ml) and allopurinol (100 μg/ml) as a positive control was measured in 96-well plates using a spectrophotometric technique previously published by Rahmi et al. (2020).

#### *In Vivo* Anti-Hyperuricemic Assay of Potassium Oxonate-Induced Rats

Only the most active EtOH80 extract (50, 100, and 200 mg/kg/day, p.o.) and AP (30 mg/kg/day, p.o.) were investigated to assess the anti-hyperuricemic effect compared to the positive control allopurinol (5 mg/kg, p.o.). The *in vivo* anti-hyperuricemic assay extract was carried out for 14 days in accordance with a previous study by Rahmi et al. (2020). Using a uric acid assay kit (BioVision, Milpitas, CA. United States), the serum uric acid levels were determined by an enzymatic-colorimetric technique on a 96-well plate (Greiner Bio One, Germany). The XO colorimetric assay kit from BioVision (Milpitas, CA, United States) was used to measure enzyme and liver XO levels.

#### Protein Expressions of Renal URAT1, GLUT9, and OAT1 in Rats by Western Blotting

Kidney samples from each treatment group were homogenized in RIPA lysis buffer with protease and phosphatase inhibitors, and chilled for 30 min. The lysate was then centrifuged at 13,000 rpm for 10 min at 4°C to extract total proteins, which were then quantified using the Bradford assay. Total proteins were incubated in boiling water for 7 min with loading buffers. On a 10% SDS-PAGE, an equal quantity of total proteins was isolated and transferred onto a PVDF membrane. Membranes were blocked in TBST containing 5% skimmed milk powder for 1 h. They were then incubated with the primary antibodies againts rabbit anti-SLC22A12 (URAT1) antibody (1: 2000, abx003918; Abbexa, UK), rabbit anti-GLUT9 antibody (1: 5,000, ab223470; Abcam, UK), rabbit anti-OAT1 antibody (1: 3,000, abx218709; Abbexa, UK), and rabbit anti- β-actin (13E5) antibody (1: 1,000, 34970; Cell Signaling, United States) in TBST containing 5% skimmed milk powder overnight at 4°C. After three TBST washes of the membranes, immunoreactive bands were identified using HRP conjugated goat anti-rabbit IgG (ab205718; Abcam, UK) as a secondary antibody that was diluted in a ratio of 1:5,000 in TBST for 1 h at room temperature. ECL detection reagent (Bio-Rad) was used to view the proteins, while ChemiDoc XRS + was used to evaluate the density of bands, which was then normalized to β-actin.

### Determination of Anti-Inflammatory Effect

#### *In Vitro* Cytokine Assay Using HFLS Cells

Human fibroblast-like synoviocyte cells (HFLS) were purchased from Cell Applications (California, United States). HFLS were grown in synoviocyte growth medium with 10% synoviocyte growth supplement in 5% CO_2_ at 37°C. Every other day, the medium was changed. When HFLS cells achieved 80% confluence, they were sub-cultured.

The MTT assay was used to determine HFLS cell viability following a method described by Rahmi et al. (2020). In short, HFLS were seeded at a density of 1 × 10^6^ per mL in 96-well plates with the same volume of extracts (i.e., EtOH80 and water), chemical markers (i.e., AP, DAP, and NAP) and dexamethasone as a positive control at concentrations of 5 and 10 μg/ml and 0.5% DMSO as a negative control (5 μg/ml), and pre-incubated for 27 h at 37°C with 5% CO_2_. After adding 20 µL of MTT reagent (5 mg/ml), the plates were incubated for an additional 4 h at 37°C with 5% CO_2_. The supernatant was discarded carefully, and the cells’ formazan blue crystals were dissolved in 100 ml DMSO. Finally, the absorbance was determined using Tecan’s Infinite 200PRO NanoQuant microplate reader at a wavelength of 570 nm.

The concentrations of IL-1α, IL-1β, IL-6, and TNF-α were measured in the HFLS culture supernatant using the ELISA technique described before (Rahmi et al., 2020). The HFLS (1 × 10^6^ per mL) were pre-treated with extracts, chemical markers and dexamethasone at a concentration of 10 μg/ml, as well as complete medium with 0.5% DMSO for 3 h at 37°C in 5% CO_2_, and then stimulated for 24 h with MSU crystal suspension (200 mg/ml). Cells were centrifuged for 10 min at 300 × g and 4°C following incubation. The supernatant was carefully transferred to a sterile tube, and the concentration of cytokines was determined using suitable human ELISA kits. The amounts of cytokine secretion were compared to a negative control, which was assumed to have 100% cytokine secretion. The IC_50_ values for the active extracts were obtained from five different concentrations (0.625–10 μg/ml).

#### Protein Expressions of MyD88, NLRP3, NF-κB, and Caspase-1 in MSU-Induced HFLS Cells by Western Blotting

Cells were seeded in 6-well plates (1 × 10^6^ cells/well) for 24 h before being pre-treated for 3 h with 1.25, 2.5, and 5 μg/ml concentrations of extracts and chemical markers. MSU crystals were then used to stimulate cells, which were then incubated for 24 h. The cells were rinsed with cold PBS and disrupted in a lysis buffer containing protease and phosphatase inhibitors for the radioimmunoprecipitation assay (RIPA). On ice, harvested cells were lysed. The lysate was then centrifuged at 13,000 rpm for 10 min at 4°C to extract total proteins, which were then quantified using the Bradford assay. Total proteins were incubated in boiling water for 5 min with loading buffers. Equal quantity of total proteins was then separated on a 10% sodium dodecyl sulfate polyacrylamide gel electrophoresis (SDS-PAGE) and transferred onto polyvinylidene difluoride (PVDF) membrane. Membranes were blocked in TBST (Tris-buffered saline with 0.1 percent Tween-20) with 5% skimmed milk powder for 1 h. They were then incubated overnight at 4°C in 3% BSA with primary antibodies from Cell Signalling, USA against MyD88 (D80F5) (1:3,000, #4283), NLRP3 (D2P5E) (1:3,000, #13158), NF-κB p-p65 (D14E12) (1:3,000, #3033), caspase 1 (D7F10) (1:3,000, #3866), and β-actin (13E5) (1:3,000, #4970S). After washing the membrane three times with TBST, immunoreactive bands were detected using a secondary antibody, anti-rabbit IgG HRP-linked antibody (Cell Signaling, United States) that was diluted in a ratio of 1:3,000 in BSA and incubated for 1 h at room temperature. ECL detection reagent (Bio-Rad) was used to visualize the proteins and ChemiDoc XRS+ was used to analyze the density of bands, which was normalized to β-actin.

#### *In Vivo* Anti-Inflammatory Assay of MSU-Induced Rats

Only the most active EtOH80 extract and AP were investigated to assess the anti-gouty inflammatory properties. An experimental model of gouty inflammation was used with MSU crystals as the inducer. The *in vivo* assay was carried out for 14 days in accordance with the previously published protocol (Rahmi et al., 2020). Briefly, 36 rats were randomly assigned to one of six experimental groups (*n* = 6 per group): normal control, hyperuricemia-induced control, indomethacin treatment (3 mg/kg/day) as a positive control, andrographolide treatment (30 mg/kg/day) and extract treatment of three different doses (50, 100, and 200 mg/kg/day). Feeding was stopped 2 h before oral administration of the allopurinol, andrographolide, or extract. Except for the normal control group, all rats were anaesthetized with isoflurane on the 11th day of the experiment, and inflammation was induced by intra-articular injection of 50 µL of MSU crystals suspension (100 mg/ml) into a rat’s right knee joint 1 h after oral treatment. A caliper was used to determine the swelling rate of a rat’s knee joint at 2, 4, 8, 12, 24, 48, and 72 h after induction. Rats were anaesthetized with isoflurane and killed by cervical dislocation at the end of experiment. After shaving the skin around the knee, 100 µL sterile saline was injected into the synovial cavity using a 27 G needle, followed by the insertion of a second needle to collect synovial fluid. Inflammation was determined by measuring the IL-1α, IL-1β, IL-6, and TNF-α levels in synovial fluid according to the manufacturer’s instructions using a multi-cytokine bead array detection system (Procarta, eBioscience, United States). The PGE_2_ level was quantified using a single ELISA assay as directed by the manufacturer. The liver, kidneys, spleen, heart, and lung were promptly removed, rinsed in cold saline (0.9%), dried with a paper towel, and weighed.

### Statistical Analysis

GraphPad Prism 5 was used to analyse all of the data and to calculate the IC_50_ values. Every experiment was performed three times (*n* = 3) in this study, and the results were presented as mean ± SEM. The test samples and controls were compared using one-way analysis of variance (ANOVA) and post hoc Tukey. If the *p* value was less than 0.05 (*p* < 0.05), the variance was considered significant.

## Results

### Quantitative Analysis of *Andrographis paniculata* Extracts Using HPLC

**FIGURE 1:**
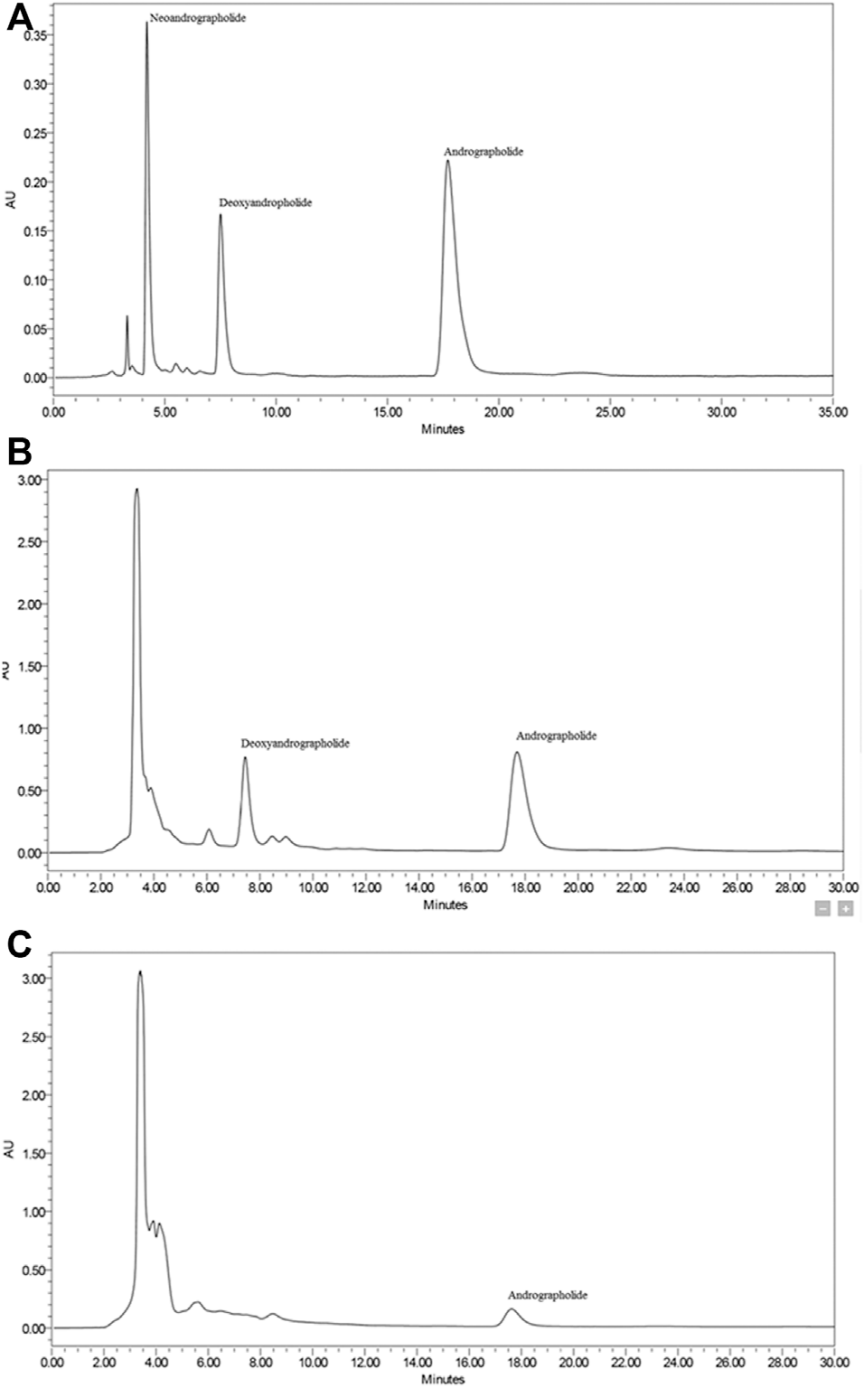
Representative HPLC chromatograms of diterpene lactones (i.e., andrographolide, 14-deoxy-11,12-didehydroandrographolide, and neoandrographolide) **(A)**, ethanol (80%) extract **(B)** and water extract **(C)** of *Andrographis paniculata* leaves.

Three diterpene lactones previously discovered in *A. paniculata* leaf extracts were detected using HPLC in this work, namely andrographolide (AP), 14-deoxy-11,12-didehydroandrographolide (DAP), and neoandrographolide (NAP) (Figure 1). Chemical markers of pure AP, DAP, and NAP were used as reference standards to identify and quantify the peaks found in the extracts. Table 1 summarizes the amount of diterpene lactones found in *A. paniculata* leaves. In general, the EtOH80 extract contained more diterpene lactones than the water extract. AP was identified at a concentration of 113.44 mg/g (11.34% w/w) in EtOH80 extract, which was greater than the concentrations of the other identifiable phytochemicals.

**TABLE 1:** Amount of andrographolide (AP), 14-deoxy-11,12-didehydroandrographolide (DAP), and neoandrographolide (NAP) in 80% of ethanol (EtOH80) and water extracts of *Andrographis paniculata* leaves analyzed by HPLC.

Standard	Concentration (mg/g)		Amount (% w/w)	
	EtOH80	Water	EtOH80	Water
AP	113.44 ± 1.63	13.788 ± 1.61	11.344 ± 0.16	1.379 ± 0.16
DAP	74.350 ± 1.88	ND	7.435 ± 0.19	ND
NAP	ND	ND	ND	ND

Data are presented as mean ± SEM (*n* = 3). ND-not detected.

### Effect of *Andrographis paniculata* Leaves on Xanthine Oxidase Inhibitory Activity *In Vitro*

Table 2 shows that all extracts and chemical markers at the tested concentrations had lower XO inhibitory activity than 50%. Thus, IC_50_ values were not determined.

**TABLE 2:** *In vitro* xanthine oxidase inhibitory activity (%) of ethanol (80%) and water extracts of *Andrographis paniculata* leaves, diterpene lactones, and allopurinol.

Test substances	% Inhibition
EtOH80 extract (400 μg/ml)	28.28 ± 2.80
Water extract (400 μg/ml)	20.71 ± 1.96
AP (100 μg/ml)	4.39 ± 0.15
DAP (100 μg/ml)	15.19 ± 1.73
NAP (100 μg/ml)	6.35 ± 0.21
Allopurinol (positive control) (100 μg/ml)	99.87 ± 0.09

Data are presented as mean ± SEM (*n* = 3). Data were analyzed by one-way ANOVA followed by post hoc Tukey. Percent inhibition >2.5% was significant at *p* < 0.05 when compared with negative control (0% inhibition). All percent inhibition values of extracts were statistically different compared with allopurinol (*p*< 0.01).

### Effect of Ethanol (80%) Extract of *Andrographis paniculata* Leaves on Serum Uric Acid Levels, Liver Xanthine Oxidase and Protein Expression of Renal URAT1, GLUT9 and OAT1 in Hyperuricemic Rats

**FIGURE 2:**
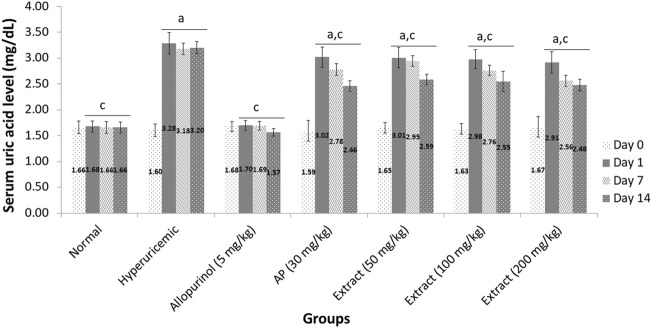
Effect of ethanol (80%) extract of *Andrographis paniculata* leaves, andrographolide and allopurinol on serum uric acid levels in hyperuricemic-induced rats. Data are presented as mean ± SEM (*n* = 6). Data were analyzed using one-way ANOVA followed by post hoc Tukey. ^a^Significantly different compared to normal group (*p* < 0.05). ^b^Not significantly different compared to normal group (*p* > 0.05). ^c^Significantly different compared to hyperuricemic group (*p* < 0.05). ^d^Not significantly different compared to allopurinol group (*p* > 0.05).

As indicated in Figure 2, the baseline serum uric acid levels of each group tested on Day 0 ranged from 1.59 to 1.68 mg/dl. When hyperuricemic rats were given EtOH80 extract at dosages of 50, 100, and 200 mg/kg for 14 days, serum uric acid levels were significantly (*p* < 0.05) decreased to 2.59, 2.54, and 2.48 mg/dl, respectively, compared to the hyperuricemic control group. However, none of the extract treatments could lower serum uric acid levels to the baseline value. When compared to the hyperuricemic control group, allopurinol (5 mg/kg) resulted in a significant (*p* < 0.05) reduction in serum uric acid levels. This decrease was seen even after 1 day of allopurinol treatment, and normal serum uric acid levels were maintained throughout the 14-days experiment with values of 1.70, 1.69, and 1.57 mg/dl on days 1, 7, and 14, respectively.

The inhibitory action of liver XO in rats was investigated to determine the antihyperuricemic efficacy of the EtOH80 extract. As demonstrated in Table 3, the hyperuricemic control group had significantly increased liver XO activity compared to the normal control group (*p* < 0.05). Treatment with EtOH80 extract at a dose of 200 mg/kg and AP reduced XO activity in the liver by only 17.01 and 19.79%, respectively, which were not comparable to the 48.61% inhibition observed with allopurinol.

**TABLE 3:** Effect of ethanol (80%) extract of *Andrographis paniculata* leaves, andrographolide and allopurinol on xanthine oxidase activity in rat’s liver.

Treatment	XO activity ± SEM (nmole uric acid/min per mg protein)	% Inhibition
Normal	2.36 ± 0.31^b^ ^,^ ^c^	—
Hyperuricemic	2.88 ± 0.42^a^ ^,^ ^c^	—
Allopurinol (5 mg/kg)	1.48 ± 0.11^a^ ^,^ ^b^	48.61 ± 0.22
Andrographolide (30 mg/kg)	2.31 ± 0.50^b^ ^,^ ^c^	19.79 ± 0.61^c^
Extract (50 mg/kg)	2.46 ± 0.42^b^ ^,^ ^c^	14.58 ± 0.58^c^
Extract (100 mg/kg)	2.42 ± 0.39^b^ ^,^ ^c^	15.97 ± 0.42^c^
Extract (200 mg/kg)	2.39 ± 0.64^b^ ^,^ ^c^	17.01 ± 0.59^c^

Data are presented as mean ± SEM (*n* = 6). Data were analysed by using one-way ANOVA followed by post hoc Tukey.

a Significantly different compared to normal group (*p* ≤ 0.05).

b Significantly different compared to hyperuricemic group (*p* ≤ 0.05).

c Significantly different compared to allopurinol (*p* ≤ 0.05).

**FIGURE 3:**
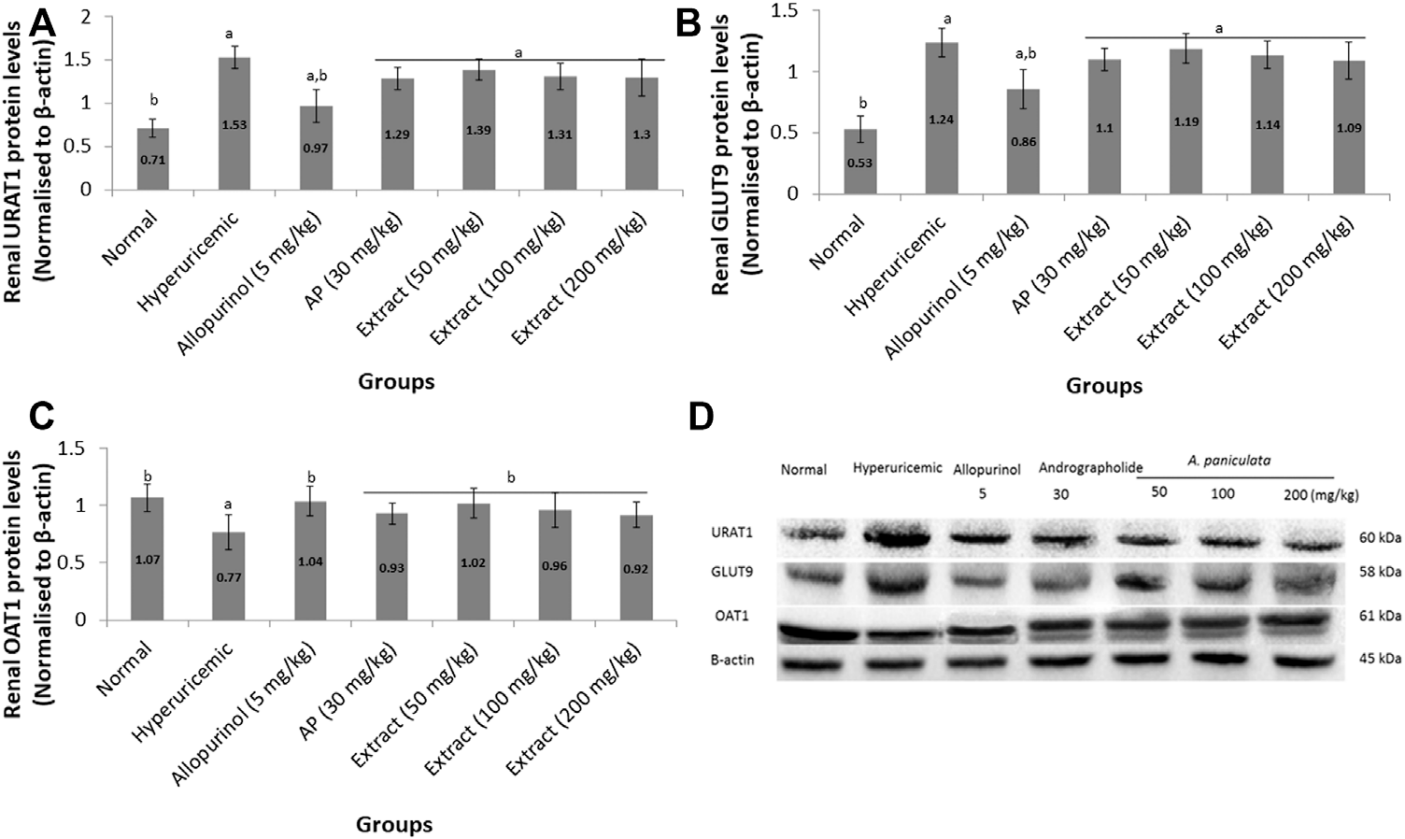
Effect of ethanol (80%) extract of *A. paniculata* leaves, andrographolide and allopurinol on protein expressions of renal URAT1 **(A)**, GLUT9 **(B)** and OAT1 **(C)** in hyperuricemic-induced rats obtained from Western blot analysis **(D)**. Data are presented as mean ± SEM (*n* = 6). Data were analyzed using one-way ANOVA followed by post hoc Tukey. ^a^Significantly different compared to normal group (*p* < 0.05). ^b^Significantly different compared to hyperuricemic group (*p* < 0.05).

Figure 3 shows the effects of EtOH80 extract, AP and allopurinol on protein expressions of URAT1, GLUT9, and OAT1 in hyperuricemic rats. The protein expression levels of renal urate transporters URAT1 and GLUT9 were reduced in potassium oxonate-induced hyperuricemic rats but were not significantly different from allopurinol. The hyperuricemic rat’s renal OAT1 protein levels were significantly lower than in the control group, which were recovered by EtOH80 extract, AP, and allopurinol.

### Effect of *Andrographis paniculata* Leaves and Diterpene Lactones on Cytokine Secretion in MSU-Induced HFLS

**FIGURE 4:**
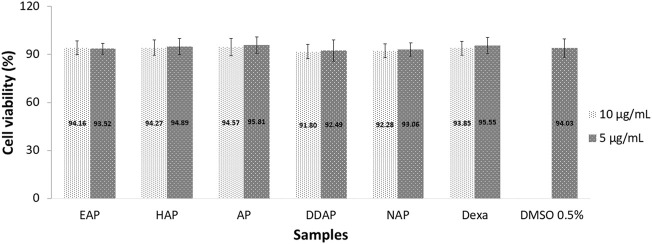
Viability of human fibroblast-like synoviocyte (HFLS) cells after 27 h of exposure to 80% ethanol (EAP) and water (HAP) extracts of *Andrographis paniculata* leaves, andrographolide (AP), 14-deoxy-11,12-didehydroandrographolide (DAP), neoandrographolide (NAP), dexamethasome (DEXA), and 0.5% DMSO. Data are presented as mean ± SEM (*n* = 3).

The MTT assay, which is based on the conversion of MTT to purple colored formazan by mitochondrial dehydrogenase from viable cells, was used to assess HFLS cell viability. More than 90% of viable cells were found at 5 and 10 μg/ml concentrations of all *A. paniculata* extracts, chemical markers, and dexamethasone, showing that such concentrations had no influence on HFLS viability after 27 h of exposure (Figure 4). As a result, in this experiment, the 10 μg/ml concentration was used as the maximum concentration. The cell viability assay was crucial in demonstrating that the inhibition of cytokine production was not related to cell death.

Table 4 summarizes the inhibitory action of EtOH80 and water crude extracts, as well as three diterpene lactones at a concentration of 10 g/ml, on IL-1α, IL-1β, IL-6, and TNF-α. Only EtOH80 extract, AP and DAP were shown to suppress cytokine release actively (> 50% inhibition). EtOH80 extract inhibited the production of four cytokines most effectively, including IL-1α (54.34%), IL-1β (79.92%), IL-6 (71.23%), and TNF-α (69.29%). Additionally, AP reduced the production of four cytokines, including IL-1α (70.45%), IL-1β (86.34%), IL-6 (74.59%), and TNF-α (72.37%). Meanwhile, DAP suppressed just two cytokines actively, namely IL-1α (50.34%) and IL-1β (50.93%). The positive control, dexamethasone effectively suppressed the production of five cytokines, i.e., IL-1α (79.36%), IL-1β (69.37%), IL-6 (70.36%), IL-8 (53.28%), and TNF-α (65.13%). However, only AP possessed a comparable inhibitory activity to dexamethasone for all cytokines (*p* ≥ 0.05). Meanwhile, EtOH80 extract possessed a comparable inhibitory activity to dexamethasone for IL-1β, IL-6 and TNF- α (*p* ≥ 0.05).

**TABLE 4:** Percentage of inhibition of ethanol (80%) and water extracts of *Andrographis paniculata* leaves, diterpene lactones and dexamethasone at a concentration of 10 μg/ml and IC_50_ values (µg/ml) on cytokine secretion in MSU-induced human fibroblast-like synoviocyte (HFLS).

Test substances and drug	% Inhibition and (IC_50_ values in μg/mL)				
	IL-1α	IL-1β	IL-6	IL-8	TNF-α
EtOH80 extract	54.34 ± 8.63	79.92 ± 7.34^a^	71.23 ± 4.21^a^	49.28 ± 3.48^a^	69.29 ± 24.12^a^
	(7.26 ± 1.21)^b^	(2.76 ± 0.23)^b^	(3.03 ± 0.69)^b^	(-)	(3.95 ± 0.97)^b^
Water extract	37.59 ± 5.46 (-)	48.23 ± 6.83 (-)	40.34 ± 3.45 (-)	33.29 ± 2.84 (-)	41.02 ± 3.48 (-)
AP	70.45 ± 7.36^a^	86.34 ± 5.34^a^	74.59 ± 4.35^a^	48.66 ± 4.92^a^	72.37 ± 5.11^a^
	(3.09 ± 1.02)^b^	(1.19 ± 0.29)^a^	(2.97 ± 0.37)^b^	(-)	(3.12 ± 1.04)^b^
DAP	50.34 ± 6.64	50.93 ± 4.35	47.88 ± 3.13	32.38 ± 4.11	44.21 ± 3.41
	(8.16 ± 1.26)^b^	(8.39 ± 1.11)^b^	(-)	(-)	(-)
NAP	44.67 ± 5.23 (-)	47.34 ± 3.25 (-)	44.63 ± 4.87 (-)	29.39 ± 2.63 (-)	38.23 ± 2.94 (-)
Dexamethasone (positive control)	79.36 ± 6.92	69.37 ± 6.14	70.36 ± 5.46	53.28 ± 3.28	65.13 ± 3.65
	(0.71 ± 0.09)	(0.57 ± 0.17)	(0.81 ± 0.16)	(0.76 ± 0.25)	(0.49 ± 0.10)

Data are presented as mean ± SEM (*n* = 3). Data were analysed using one-way ANOVA followed by post hoc Tukey. Percentage inhibition >2.5% was significant at *p* ≤ 0.05 when compared with negative control. (-) = IC_50_ was not determined as none of tested concentration exceeded 50% inhibition.

a Not significantly different compared to dexamethasone (*p* ≥ 0.05).

b Significantly different compared to dexamethasone (*p* < 0.01).

### Effect of *Andrographis paniculata* Leaves and Diterpene Lactones on Protein Expressions of MyD88, NLRP3, NF-κB and Caspase-1 in MSU-Induced HFLS Cells

**FIGURE 5:**
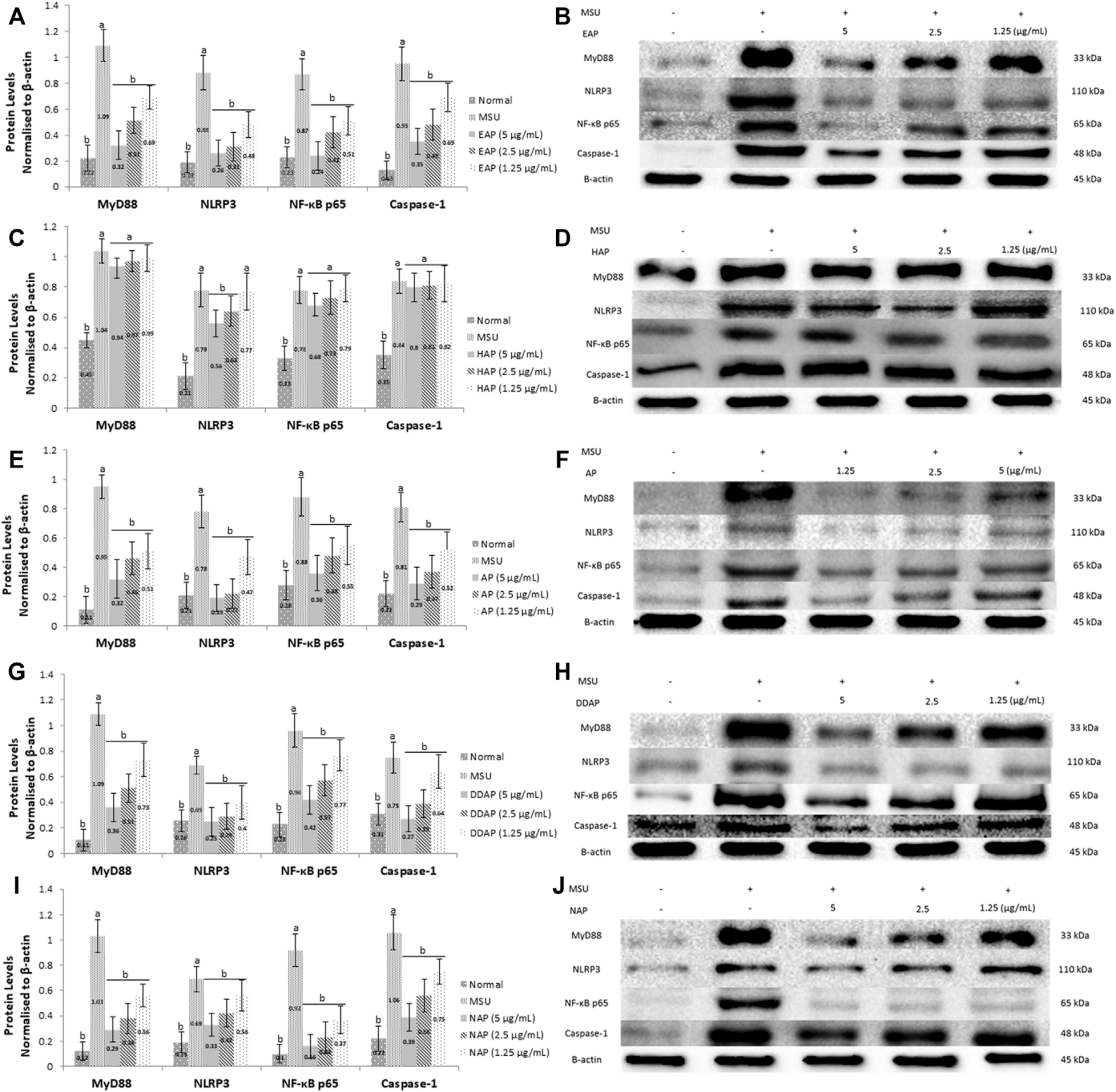
Effect of ethanol (80%) extract **(A,B)**, water extract **(C,D)** of *A. paniculata* leaves, andrograpolide **(E,F)**, 14-deoxy-11,12-didehydroandrographolide **(G,H)** and neoandrographolide **(I,J)** on protein expressions of MyD88, NLRP3, NF-κB, and caspase-1 in MSU-induced HFLS. Data are presented as mean ± SEM (*n* = 3). Data were analyzed using one-way ANOVA followed by post-hoc Tukey. ^a^Significantly different compared to normal group (*p* < 0.05). ^b^Significantly different compared to MSU-induced group (*p* < 0.05).

To elucidate the mechanisms of action by which *A. paniculata* and its main constituents reduced MSU-induced inflammation in HFLS, western blot analysis was used to determine the MyD88, NLRP3, NF-κB, and caspase-1 protein levels. The results (Figure 5) demonstrated that MSU-induced HFLS significantly (*p* < 0.05) increased protein expression levels for all protein targets when compared to untreated cells. The treatment of EtOH80 extract, AP, DAP, and NAP significantly (*p* < 0.05) reduced the expression of all target proteins following MSU induction in a concentration dependent manner. However, treatment with water extract had no significant effect on the expression of MyD88, NF-κB, or caspase-1 in MSU-induced HFLS. In fact, it reduced NLRP3 expression considerably only at concentrations of 5 and 2.5 μg/ml.

### Effect of *Andrographis paniculata* Leaf Ethanol (80%) Extract and Andrographolide on Swelling Rate, Cytokine Secretion and PGE_2_ Secretion of MSU-Induced Inflammation in Rats

**FIGURE 6:**
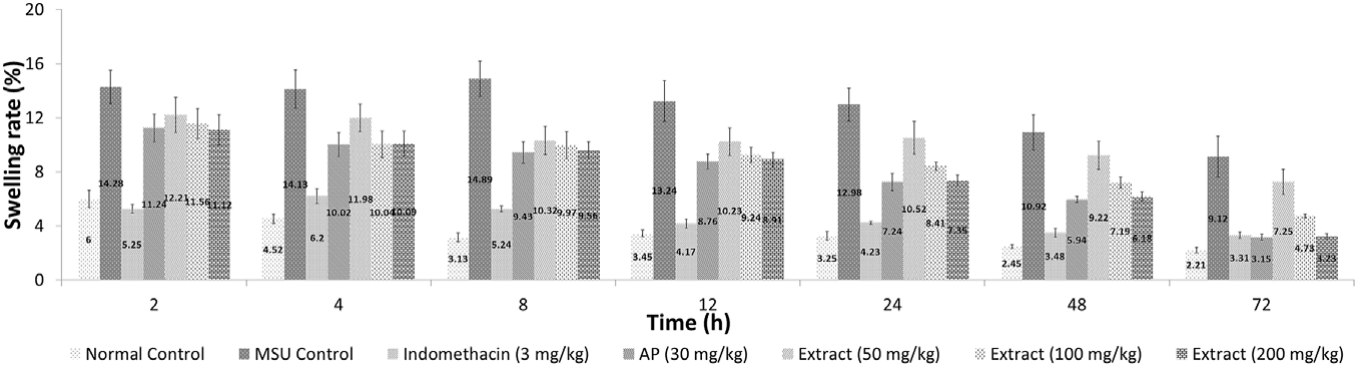
Effect of ethanol (80%) extract of *Andrographis paniculata* leaves, andrographolide, and indomethacin on swelling rate in MSU-induced inflammation in rat’s knee joint.

**FIGURE 7:**
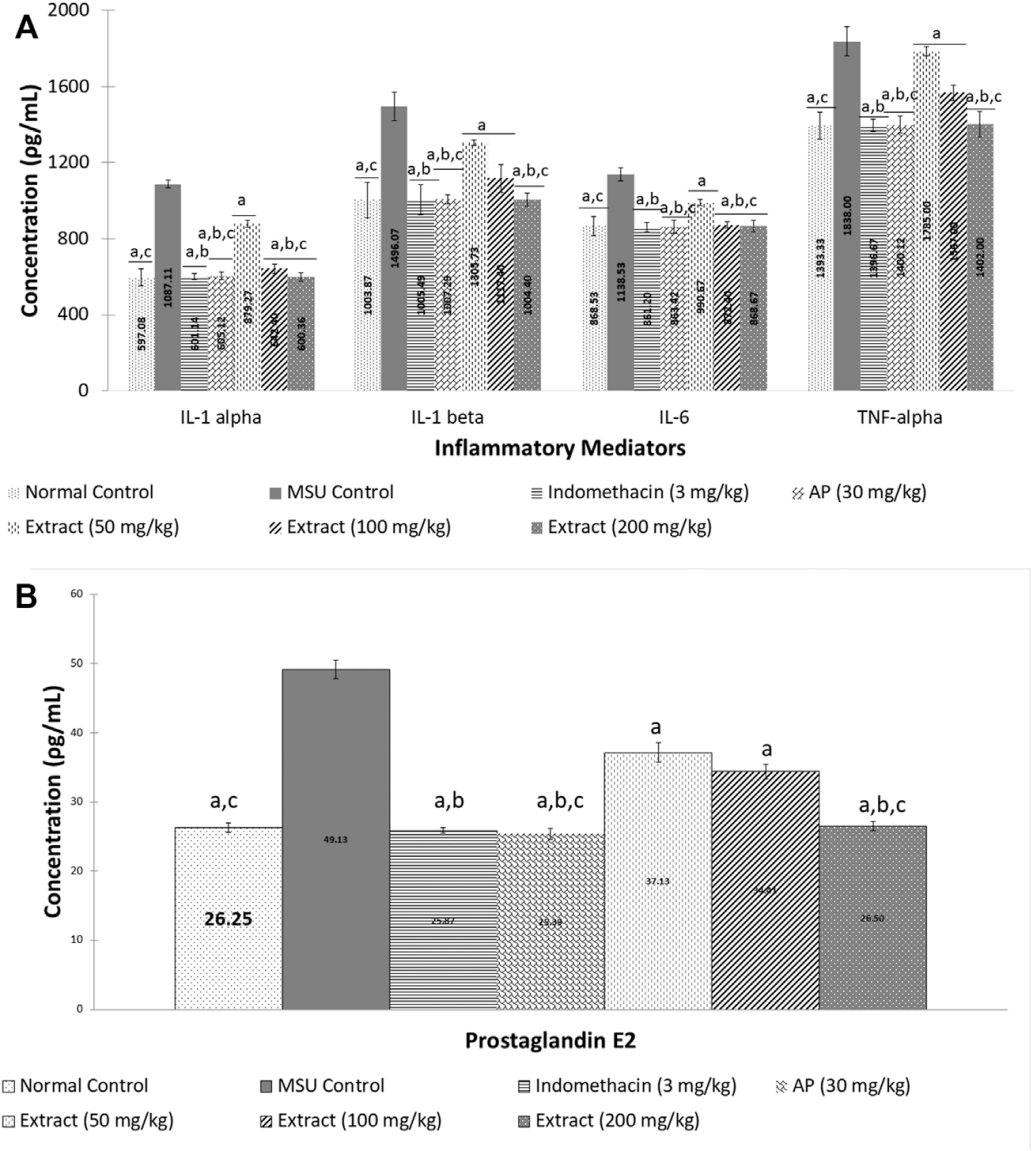
Effect of ethanol (80%) extract of *Andrographis paniculata* leaves and andrographolide on MSU-induced inflammatory mediator secretion in rat’s knee joint synovial fluid: **(A)** cytokines and **(B)** prostaglandin E_2_. Data are presented as mean ± SEM (*n* = 6). ^a^Significantly different compared to MSU control group (*p* < 0.05). ^b^Not significantly different compared to normal control group (*p* > 0.05). ^c^Not significantly different compared to indomethacin group (*p* > 0.05).

After 72 h, EtOH80 extract (200 mg/kg) and AP (30 mg/kg) reduced swelling to normal levels, equivalent to the indomethacin (3 mg/kg) group (Figure 6). In addition, treatment with EtOH80 extract (200 mg/kg) and AP (30 mg/kg) significantly (*p* < 0.05) decreased levels of IL-1α, IL-1β, IL-6, TNF-α, and PGE_2_ in a dose-dependent manner, equivalent to indomethacin and normal control groups (*p* > 0.05) (Figure 7).

### Body and Organ Weight Observation

During the 14-days experiment, changes in body weight (Supplementary Figure S1) and organ weight (Supplementary Tables S1, S2) were noted. The findings revealed that there were no significant changes in body and organ weight for any of the groups when compared to their baseline values (*p* > 0.05). Physical observations revealed no changes in the rats’ skin, hair, eyes, mucous membranes, behavioral pattern, tremor, salivation, or diarrhea. There was no death or substantial weight loss in the rats at the tested dosages (*p* > 0.05).

## Discussion

In Indonesia and Malaysia, *A. paniculata* is a widely used traditional medicine. Despite its unpleasant bitter taste, the plant is often consumed as a herbal drink and fresh salad among other vegetables. *A. paniculata* has been extensively used empirically to treat a variety of illnesses, including inflammation, allergic reactions, and as an immunomodulator. Numerous publications have reported on the anti-inflammatory effects of *A. paniculata* and its bioactive components. Although *A. paniculata* has many anti-inflammatory chemicals, andrographolide is the most prevalent (Dong et al., 2009; Parichatikanond et al., 2010).

Gout develops because of the crystallization of uric acid in tissues, tendons, and joints, triggering inflammatory reactions (Chen et al., 2011). Uric acid is a byproduct of human purine metabolism that is, insoluble in blood. When uric acid levels exceed 7 mg/dl, the physiologic fluid becomes saturated, resulting in crystal formation in joints, tendons, and tissues. This syndrome hastens the onset of gout, which is characterized by repeated attacks of acute inflammation (Nuki 2006).

MSU crystals can initiate, intensify, and maintain an inflammatory attack by promoting the production and release of humoral and cellular inflammatory mediators (Choi et al., 2005). Toll-like receptor 2/4 (TLR 2/4) and CD16, which are components of the innate immune system, are the first to recognize MSU crystals. By binding with membrane antigen-presenting cells (APCs), it stimulates the spleen tyrosine kinase (Syk) pathway, causing lipid sorting and the aggregation of immunoreceptor tyrosine-based activation motifs (ITAMs). The downstream of phosphoinositide 3-kinases (PI3Ks), which are crucial in phagocytosis, is then activated by Syk. Phagocytosis causes lysosomal damage and activates the inflammasome via two distinct signaling pathways: 1) a signal from Syk activates pro-IL-1β production *via* CARD9, while ROS production and K+ efflux activates the NLRP3 inflammasome and leads to IL-1β activation; and 2) a signal from TLR activates NF-κB, which activates pro-IL-1β production *via* the MyD88/TRIF pathway. MSU phagocytosis results in cathepsin B leaking into the cytoplasm, which activates the inflammasome. Finally, non-hematopoetic cells or IL-1R produce mature IL-1β as an inflammatory mediator (Shi et al., 2012).

When MSU crystals interact with cells, particularly monocytes, they upregulate IL-6 and TNF-α secretion (Busso and So 2010). In addition, chemokines including IL-8 (CXCL8) and cyclooxygenase-2 (COX-2) have a role in gouty inflammation (Choi et al., 2005). The action of the cyclooxygenase-2 (COX-2) enzyme and PGE_2_ synthase in endothelial cells biosynthesizes prostaglandin E_2_ (PGE_2_) from arachidonic acid precursor. PGE_2_ plays a crucial role as a pro-inflammatory mediator in inflammation. As a result, a PGE_2_ production inhibitor might be useful as a treatment for inflammation (Ward, 2010). One of the modes of action of inflammation in gout is the production of eiconasoids, particularly PGE_2_, *via* MSU crystals formation (Joosten et al., 2010). Various inflammatory mediators, such as cytokine and prostanoids, are produced when MSU crystals are stimulated in joint tissues. The inflammatory characteristics of intense pain, oedema, and erythema in the joints are all caused by these mediators. Prostanoids, particularly PGE_2_, are essential for the development of pain, vasodilation, oedema, and leukocyte migration. According to Pauliot et al. (1998), MSU crystals increased COX-2 production in monocytes, which was linked to PGE_2_ and tromboxaneA_2_ (TXA_2_) secretion. As a result, inhibiting PGE_2_ production is crucial in the therapy of gouty inflammation.

The kidneys excrete uric acid with the aid of uric acid transporters. Recent studies have demonstrated that hyperuricemia is related with alterations in the expression and function of urate transporters. Uric acid transporters are classified into two types: those that promote urate reabsorption, such as urate anion transporter 1 (URAT1), organic anion transporter 4 (OAT4), and glucose transporter 9 (GLUT9), and those that promote urate excretion, such as OAT1, OAT3, urate transporter (UAT), multidrug resistance protein 4 (MRP4/ABCC4), ABCG-2, and sodium-dependent phosphate transport protein.

The effects of *A. paniculata* extracts and its bioactive compounds, such as andrographolide, 14-deoxy-11,12-didehydroandrographolide, and neoandrographolide, on anti-gout activity in terms of lowering uric acid and reducing crystal-induced inflammation were investigated in this study. The anti-inflammatory effect of this study was focused on the MyD88 pathway, which regulates the synthesis of NLRP3, NF-κB, caspase-1, and IL-1β. Meanwhile, the anti-hyperuricemic effect was attributed to its ability to modulate uric acid transporters, including URAT1, GLUT9, and OAT1.

Our findings indicated that 80% ethanol extract of *A. paniculata* and one of its primary constituents, andrographolide, inhibited expression of MyD88, NLRP3, NF-κB, and caspase-1 proteins in MSU-induced HFLS. Additionally, the extract and andrographolide reduced MSU-induced cytokine production of IL-1α, IL-1β, IL-6, and TNF-α in HFLS and hyperuricemic rats, as well as PGE_2_ in rats. Furthermore, the extract and andrographolide decreased the protein expression levels of the renal urate transporters URAT1 and GLUT9 in hyperuricemic rats induced by potassium oxonate, but the reduction was not comparable to that of allopurinol. Meanwhile, renal OAT1 protein levels were considerably reduced in potassium oxonate-induced hyperuricemic rats compared to the normal control rats, which were significantly recovered by the extract, andrographolide and allopurinol.

## Conclusion

Overall, the results indicated that an ethanol (80%) extract of *A. paniculata* leaves and its main diterpene lactone compound, andrographolide, may have antihyperuricemic and anti-inflammatory properties and therefore be used to treat gout. *In vivo*, the antihyperuricemic action was mediated by xanthine oxidase inhibition or maybe in synergy with other mechanisms such as uricosuric effect. Meanwhile, anti-inflammatory action was demonstrated by inhibitory activity of monosodium urate crystals-induced cytokines and prostaglandin E_2_ secretion. *In vitro* assays with HFLS may indicate that EtOH extract of *A. paniculata* leaves and andrographolide inhibited gouty inflammation by inhibiting the production of cytokines and protein expression of MyD88, NLRP3, NF-κB, and caspase-1. This research establishes a foundation for the development of a novel anti-inflammatory phytotherapy. It is believed that well characterized standardised *A. paniculata* herbal product gives an excellent opportunity to further investigate its potential treatment of gouty-inflammatory conditions.

## Data Availability

The original contributions presented in the study are included in the article/Supplementary Material, further inquiries can be directed to the corresponding author.
