# Supplementary material for: Extracts of Andrographis paniculata (Burm.f.) Nees Leaves Exert Anti-Gout Effects by Lowering Uric Acid Levels and Reducing Monosodium Urate Crystal-Induced Inflammation
Source: Front Pharmacol. 2022 Jan 10;12:787125. doi: 10.3389/fphar.2021.787125 (PMC8793851; doi:10.3389/fphar.2021.787125)
Supplement: Supplementary file 1 [file DataSheet1.docx]

Supplementary Material

# Supplementary Figures and Tables

The body weight observation did not show significant differences among groups during the 14 experimental days (Supplementary Figure 1), although a slight non-significant body weight decrease occurred with allopurinol and extracts. Furthermore, proportional weight of liver, kidney, spleen, heart, and lung did not differ among group (Supplementary Table 1 and 2).

## Supplementary Figures


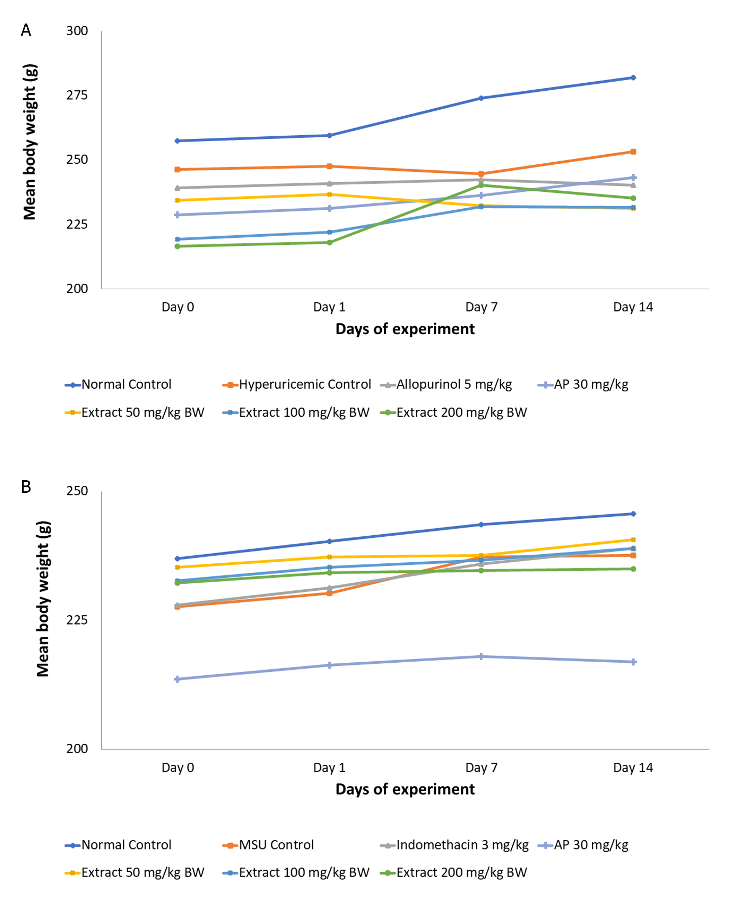


**Supplementary Figure 1.** Body weight monitoring before drug dosing (day 0) and on days 1, 7 and 14 for the anti-hyperuricemic (A) and anti-inflammatory (B) experiments. Data were analyzed using one-way ANOVA and followed by post hoc Tukey. No significant difference observed between day 1, day 7, day 14 vs. respected day 0 (*p*>0.05)

## Supplementary Tables

Supplementary Table 1 Index organ of rats after 14 days of experiment using ethanol (80%) extract of *Andrographis paniculata* leaves, andrographolide and allopurinol in anti-hyperuricemic *in vivo* assay

| Animal Groups | Index organ (%) | | | | | |
| --- | --- | --- | --- | --- | --- | --- |
|  | Liver | Kidneys | Spleen | Heart | Lung | Stomach |
| Normal Control | 2.76 ± 0.06 | 0.63 ± 0.01 | 0.23 ± 0.01 | 0.29 ± 0.01 | 0.56 ± 0.01 | 0.49 ± 0.01 |
| Hyperuricemic Control | 3.42 ± 0.06 | 0.64 ± 0.01 | 0.28 ± 0.01 | 0.31 ± 0.01 | 0.54 ± 0.02 | 0.52 ± 0.01 |
| Allopurinol  (5 mg/kg) | 3.40 ± 0.17 | 0.71 ± 0.02 | 0.26 ± 0.01 | 0.31 ± 0.01 | 0.55 ± 0.02 | 0.48 ± 0.03 |
| Andrographolide (30 mg/kg) | 3.41 ± 0.05 | 0.68 ± 0.01 | 0.22 ± 0.03 | 0.33 ± 0.01 | 0.51 ± 0.01 | 0.55 ± 0.02 |
| Extract  (50 mg/kg) | 2.87 ± 0.03 | 0.60 ± 0.01 | 0.19 ± 0.02 | 0.30 ± 0.01 | 0.46 ± 0.03 | 0.50 ± 0.03 |
| Extract  (100 mg/kg) | 3.05 ± 0.16 | 0.62 ± 0.03 | 0.15 ± 0.01 | 0.30 ± 0.01 | 0.44 ± 0.02 | 0.48 ± 0.01 |
| Extract  (200 mg/kg) | 2.92 ± 0.07 | 0.58 ± 0.01 | 0.20 ± 0.03 | 0.29 ± 0.01 | 0.54 ± 0.01 | 0.47 ± 0.01 |

Data are presented as mean ± SEM (n=6). Data were analyzed using one-way ANOVA followed by post hoc Tukey. Relative organ weight was calculated as (organ weight/body weight) x 100%. No significant difference between treatment group vs. normal group (*p*>0.05).

Supplementary Table 2 Index organ of rats after 14 days of experiment using ethanol (80%) extract of *Andrographis paniculata* leaves, andrographolide and indomethacin in anti-inflammatory *in vivo* assay

| **Animal Groups** | **Index organ (%)** | | | | | |
| --- | --- | --- | --- | --- | --- | --- |
|  | **Liver** | **Kidneys** | **Spleen** | **Heart** | **Lung** | **Stomach** |
| Normal Control | 2.37 ± 0.19 | 0.61 ± 0.02 | 0.22 ± 0.08 | 0.30 ± 0.02 | 0.48 ± 0.02 | 0.57 ± 0.02 |
| MSU Control | 2.43 ± 0.11 | 0.62 ± 0.02 | 0.18 ± 0.03 | 0.30 ± 0.01 | 0.50 ± 0.02 | 0.59 ± 0.02 |
| Indomethacin  (3 mg/kg) | 2.55 ± 0.06 | 0.70 ± 0.02 | 0.19 ± 0.03 | 0.31 ± 0.01 | 0.49 ± 0.03 | 0.56 ± 0.05 |
| Andrographolide (30 mg/kg) | 2.64 ± 0.08 | 0.63 ± 0.01 | 0.18 ± 0.02 | 0.32 ± 0.02 | 0.55 ± 0.03 | 0.55 ± 0.01 |
| Extract  (50 mg/kg) | 2.47 ± 0.08 | 0.63 ± 0.01 | 0.18 ± 0.03 | 0.38 ± 0.07 | 0.41 ± 0.05 | 0.60 ± 0.04 |
| Extract  (100 mg/kg) | 2.45 ± 0.08 | 0.64 ± 0.01 | 0.14 ± 0.01 | 0.29 ± 0.01 | 0.49 ± 0.01 | 0.57 ± 0.02 |
| Extract  (200 mg/kg) | 2.57 ± 0.13 | 0.67 ± 0.06 | 0.18 ± 0.02 | 0.30 ± 0.01 | 0.50 ± 0.01 | 0.63 ± 0.01 |

Data are presented as mean ± SEM. Data were analyzed using one-way ANOVA followed by post hoc Tukey. Index organ was calculated as (organ weight/body weight) x 100%. No significant differences between treatment group vs. normal group.
